# Identification of Specific Hydroxyapatite {001} Binding Heptapeptide by Phage Display and Its Nucleation Effect

**DOI:** 10.3390/ma9080700

**Published:** 2016-08-17

**Authors:** Jing Mao, Xin Shi, Ya-Bo Wu, Shi-Qiang Gong

**Affiliations:** Department of Stomatology, Tongji Hospital, Tongji Medical College, Huazhong University of Science and Technology, Wuhan 430030, China; maojing1999@yahoo.com (J.M.); 13667219691@163.com (X.S.); wuyabotjh@163.com (Y.-B.W.)

**Keywords:** biomimetic, biomineralization, hydrxyapatite, solid-binding peptide

## Abstract

With recent developments of molecular biomimetics that combine genetic engineering and nanotechnology, peptides can be genetically engineered to bind specifically to inorganic components and execute the task of collagen matrix proteins. In this study, using biogenous tooth enamel as binding substrate, we identified a new heptapeptide (enamel high-affinity binding peptide, EHBP) from linear 7-mer peptide phage display library. Through the output/input affinity test, it was found that EHBP has the highest affinity to enamel with an output/input ratio of 14.814 × 10^−7^, while a random peptide (RP) displayed much lower output/input ratio of 0.00035 × 10^−7^. This binding affinity was also verified by confocal laser scanning microscopy (CLSM) analysis. It was found that EHBP absorbing onto the enamel surface exhibits highest normalized fluorescence intensity (5.6 ± 1.2), comparing to the intensity of EHBP to enamel longitudinal section (1.5 ± 0.9) (*p* < 0.05) as well as to the intensity of a low-affinity binding peptide (ELBP) to enamel (1.5 ± 0.5) (*p* < 0.05). Transmission electron microscopy (TEM), Attenuated total Reflection-Fourier transform infrared spectroscopy (ATR-FTIR), and X-ray Diffraction (XRD) studies further confirmed that crystallized hydroxyapatite were precipitated in the mineralization solution containing EHBP. To better understand the nucleation effect of EHBP, EHBP was further investigated on its interaction with calcium phosphate clusters through in vitro mineralization model. The calcium and phosphate ion consumption as well as zeta potential survey revealed that EHBP might previously adsorb to phosphate (PO_4_^3−^) groups and then initiate the precipitation of calcium and phosphate groups. This study not only proved the electrostatic interaction of phosphate group and the genetically engineering solid-binding peptide, but also provided a novel nucleation motif for potential applications in guided hard tissue biomineralization and regeneration.

## 1. Introduction

Hydroxyapatite (HAp) is the most stable form of calcium phosphate and the primary inorganic component of natural hard tissues including bone and tooth both in vertebrate animals and human [1]. It has excellent physiochemical and biological properties and integrates uniquely with organic components [2]. The subtle difference of HAp structure from nanoscale to macroscale accounts for the significant variations in mechanical properties, the diversity of hard tissues, and its applications in biomedical fields as well [3,4,5]. For example, HAp found in teeth is more highly assembled into parallel arrays than typically found in bone, resulting in robust mechanical behavior and improved acid-resistance property [6]. It is generally believed that the nucleation, crystallography, polymorphism, and morphology of HAp are precisely controlled through specific interactions between the macromolecules and inorganics [7,8,9,10,11,12]. Recently, it was demonstrated that HAp formation does not occur directly by the associated ions (i.e., classical mineralization theory) but proceeds through amorphous calcium phosphate (ACP) precursors in mineralizing environments (i.e., non-classic mineralization theory) [13]. Although ACP precursors are undetectable by conventional analytical techniques, they are postulated as basic unit of HAp crystals [13,14]. As described by non-classical mineralization model, the crystal nucleation starts with the calcium phosphate pre-nucleation clusters at the template surface, followed by densification of these clusters and phase transition into crystalline apatite [15,16]. The most important event for the hierarchical mineral biomass formation is the subtly self-assembly process of apatite/macromolecular hybrid building block [17,18]. Many efforts have been devoted into controlling the programmed precipitation of HAp into the desired space. For example, acidic non-collagenous proteins (NCPs) serve as nucleator for ACP precipitation and align ACP in an orientation that facilitates the formation of HAp crystal lattice [19]. In other studies, the calcium-enriching additives as well as some polymers have been used as stabilizer or inhibitor to encourage the infiltration of ACP precursors into the fibrillar collagen [20,21,22]. In addition, collagen was biomimetically carboxymethylated to increase the local concentration of corresponding ions so that a critical nucleus of ions can be formed, leading to the formation of the mineral. Thus, the self-organization of HAP nanocrystals on and within collagen fibrils was intensified by carboxymethylation of collagen [23,24,25]. Despite extensive investigation, the mechanism of biological mineralization process remains largely unknown.

Biomimetic mineralization (i.e., biomienralization) of biological matrices in vitro represents a significant progress towards developing organic/inorganic hybrid materials for human mineralized tissue repair [26]. Reconstruction of bone or tooth based on biomineralization principles that employ the HAp-binding peptides to facilitate the HAp crystal nucleation, growth, and arrangement through the interfacial recognition appeared to be a practical strategy. Except for the known natural proteins and motifs [27,28], genetically engineering peptides for inorganics (GEPIs) isolated and designed at the molecular level through phage display methodology are evolutionary and rationalized peptide sequences for HAp-binding [29,30,31,32]. The GEPIs from the inorganic materials surfaces are capable of nucleating, orientating and arranging the inorganic nanoparticles or providing molecular scaffolds [33,34,35,36]. Many studies have employed the phage display method to identify peptides on HAp disks or particles and investigated their in vitro specific binding affinities [37,38]. For the synthesized HAp disks or particles, the exposed crystal faces are mainly composed of {100} and are anisotropic due to the disordered array of the polycrystalline. It is well known that the proteins and peptides interact with inorganic crystals (e.g., calcite, octacalcium phosphate, and apatite) in a face-specific manner [39]. Therefore, the irregular array of crystal might compromise specificity of bacteriophage binding to the intended crystal face [40,41,42]. The outer surface of enamel of tooth is composed of predominant HAp {001} crystal faces by an assembly of the apatite nanoparticles along the *c*-axis direction, which makes it an ideal substrate for screening GEPIs with face-specific affinity to HAp crystals [43,44]. Our previous study of solid surface binding peptide to titanium bulks also incites us to acquire the HAp-binding sequence against enamel surface of desired {001} crystal face [45]. In this study, we have identified a heptapeptide which specifically binds to HAp {001} face and is responsible for nucleation and growth of HAp crystals in vitro.

## 2. Experimental Section

### 2.1. Identification of Enamel Binding Peptides

Caries-free premolars extracted for orthodontic reasons were used in this study with the patient’s informed consent and approved by the ethics committee of Huazhong University of Science and Technology (Approval number TJ-C20150314). The tooth samples were removed off any attached soft tissue, ultrasonically cleaned, sand-blasted under 0.1 MPa pressure, and treated in 5.25% sodium hypocholorite (NaOCl) for 30 s to remove the protein. After completely rinsing with distilled deionized water (dd H_2_O), the tooth samples were sterilized under ultraviolet light for 30 min before use. The Ph.D.-7™ phage display peptide library kit consisting of about 2.8 × 10^9^ different phages with 7-mer amino acid linear peptide inserts was purchased from New England Biolabs (NEB; Ipswich, MA, USA. Https://www.neb.com/). Peptides with preferential binding affinity to enamel surface were identified by screening the phage library against tooth enamel surface. Biopanning procedure has been previously described [45], complying with the protocol in detail in instruction manual attached to the kit. Only the tooth crown was immersed into the phage pool for biopanning. After each round of biopanning, the enamel surface was re-deproteinized with 5.25% NaOCl, as described above.

Besides a random monoclone from the original library, 31 monoclones were harvested from the 4th biopanning round, and named E-1 to E-32, respectively. The binding affinity of these monoclones were assayed and expressed as the ratio of the output phage number (number of phages that strongly bind to enamel) to the input phage number (number of phages invested). According to the result of binding assay, a high binding affinity sequence, NNHYLPR (expressed on monoclone E-32, named afterwards as EHBP, enamel high-affinity binding peptide), a low binding affinity sequence, GQAGERK (expressed on monoclone E-6, named as ELBP, enamel low-affinity binding peptide), and a sequence randomly selected from the original library, WGNYAYK (expressed on monoclone E-1, denoted as RP, random peptide), as well as the FITC-labeled EHBP, ELBP and RP were synthesized using the solid phase peptide synthesis (SPPS) method, and purified by HPLC and identified by amino acid analysis for further study. The lyophilized peptides were reconstituted in sterile deionized water to a final concentration of 100 μg/mL.

### 2.2. Binding Specificity of EHBP to Enamel Surface

Enamel blocks and longitudinal sections of tooth were cut from sound premolars using a cutting machine (Isomet 500, Buehler, Lake Bluff, IL, USA) under water cooling to prevent overheating. Here, “enamel blocks” mentioned contained an occlusal surface that was used for peptide binding and the “longitudinal sections” were pieces of enamel cut perpendicular to the occlusal surface. The enamel block surfaces were treated as mentioned previously and then incubated overnight at 4 °C with FITC-labeled EHBP or ELBP at the concentration 0.4 mM, respectively (pH 7.4). The excess and non-binding peptides were removed by washing three times with tris-buffered saline containing 0.1% tween-20 (TBS-T, pH 7.4). The FITC-labeled peptides that remained bound to the surface were mounted in 90 vol % of glycerol diluted in 10 mM PBS (pH 7.4) and visualized using the CLSM. Fluorescence intensity was calculated using Image–Pro Plus 6.0 software (Media Cyberhetics Inc., Bethesda, MD, USA) based on 12 non-overlapping region of interest (N = 12). Binding affinity of the two peptides to enamel surface or longitudinal section was assessed by normalizing fluorescence intensity to that of respective control group with 0.4 mM FITC without any conjugate. Two way analyses of variances (ANOVA) were performed based on two factors: One factor was peptide (EHBP or ELBP) and the other was substrate (enamel surface or longitudinal section).

### 2.3. Characterization of the Deposits of in Vitro Mineralization

Freshly prepared mineralization solution containing 2.5 mM CaCl_2_ and 5 mM Na_2_HPO_4_ was buffered by 25 mM Tris-HCl to pH 7.4. The mineralization solution was further incubated at 37 °C, 4 h after 0.4 mM EHBP added, then centrifuged at 4000 rpm for 10 min. The precipitate was collected and washed with dd H_2_O for three times to remove the excess soluble mineral salt. Mineral formed in the mineralization solution with ELBP, as well as RP at the same concentration was used as comparison. The mineral precipitate dispersed in dd H_2_O was dipped onto the carbon-coated 400-mesh Ni grids (EMS, Hatfield, PA, USA) and air-dried. The grids were examined by TEM (CM 12, Philips, Eindhoven, The Netherlands) at 110 keV as well as selected area electron diffraction (SAED) to determine the nano-architecture and crystal phase.

The in vitro mineralizing precipitates were also examined by attenuated total reflection-Fourier transform infrared spectroscopy (ATR-FTIR) to characterize the mineral phase transformation during the induction of mineral formation by bimacromolecules [33]. Infrared spectra were recorded from 4000 cm^−1^ to 400 cm^−1^ using a Fourier-transform infrared spectrometer (VERTEX 70, Bruker optics, Ettlingen, Germany) with an attenuated total reflection (ATR) at a resolution of 2 cm^−1^ and averaging 16 scans per spectrum. X-ray diffraction (Rigaku America, Woodlands, TX, USA) of the mineralizing precipitates was performed using Ni-filtered Cu Kα radiation (30 KeV, 20 mA), in the 2θ range of 5°–75°, with a scan rate of 4° per min, and a sampling interval of 0.02°.

### 2.4. Kinetic Study of in Vitro Induced Mineralization

To better understand the mechanism of EHBP induced mineralization, the calcium and phosphate ion consumption was used to monitor the kinetic of HAp nucleation. The mineralization solution containing 2.5 mM Ca^2+^ and 5 mM Na_2_HPO_4_ was prepared in 25 mM Tris-HCl buffer (pH 7.4) in sterile tubes to serve as a model system that forms HAp through calcium phosphate clusters [46]. Four groups of mineralization were classified as follows: (1) Blank group without any peptide; (2) RP group with 0.4 mM RP; (3) ELBP group with 0.4 mM ELBP; and (4) EHBP group with 0.4 mM EHBP. The mineralization solution was tightly sealed in tubes and incubated at 37 °C. Mineral formation was monitored by periodic assays of calcium and phosphate ions throughout the reaction.

For assays of calcium and phosphate, mineralization solution was collected at interval times. Colorimetric method was employed to measure calcium and phosphate ion concentrations in the mineralization processes. We used QuantiChrom™ Calcium Assay Kit (DICA-500, BioAssay Systems, Hayward, CA, USA) to measure the free calcium ions during the mineralization process. By forming a very stable blue colored complex between a phenolsulphonephtalein dye and free calcium ions, the kit can detect calcium concentration from 20 μM to 5 mM, using as little as 5 μL samples. QuantiChrom™ Phosphate Assay Kit (DIPI-500, BioAssay Systems, Hayward, CA, USA) that was based on quantification of the stable green complex between malachite green dye and free orthophosphate was used to measure phosphate ion concentration. It can measure phosphate in a linear detection range from 0.02 μM to 40 μM, using 50-μL samples. Therefore, the samples (contain ~2.6–3.0 mM phosphate) were diluted by 100 times before phosphate determination. Calcium and phosphate ion concentrations in mineral precipitations were calculated to monitor the kinetic of HAp nucleation using the assay kit.

To determine the electrostatic interaction between the bimolecular and calcium phosphate clusters in the solution, Zeta potential as well as particle size distribution was collected using the Zetaplus analyzer (Brookhaven Instruments, New York, NY, USA). EHBP at different concentration (0 mM, 0.1 mM, 0.2 mM, 0.4 mM) was completely mixed with the aforementioned mineralization solution for 1 h. Analysis of the nanoparticles suspended in the solution was performed in duplicates using a flow cell at 25 °C. The zeta potential of charged particles in solution was determined by the electrophoretic light scattering method.

## 3. Results and Discussion

**Figure 1:**
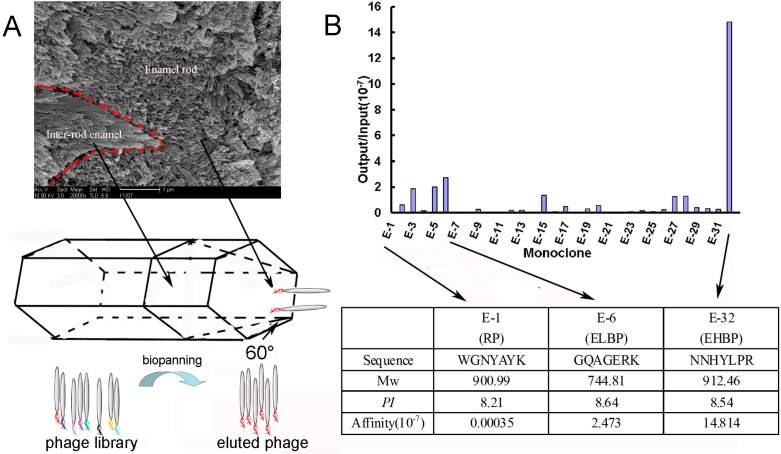
(**A**) FSEM observation of enamel surface after deproteinization showed the enamel rod and inter-rod enamel ((**A**), upper), which was schemed in (**A**), middle. The lower in (**A**) describes the procedure of biopanning from phage pool against the enamel surface; (**B**) 32 monoclones of phage from the original pool and the fourth-round panning was chosen to determine their affinity against the enamel surface through output/input assay. The peptide sequences and physicochemical properties of RP (random phage from phage pool), ELBP (low affinity to enamel) and EHBP (high affinity to enamel) displayed by selected cloned phages E-1, E-6 and E-32, respectively.

As it is well known that the outer enamel surface is composed of numerous {001} faces by an assembling of the apatite along the *c* direction, after microblasting and deproteinization, the enamel {001} faces were exposed and used as reacting substrate for phage biopanning. Figure 1A illustrates the procedure of activating the enamel surface and obtaining the binding peptide from the phage display system against the enamel {001} faces. Although {100} is the largest surface of the typical HAp crystal, it is {001}, the smallest habit face, that is chosen by the living organisms to build the outer surface of enamel by an oriented assembly of the rod-like crystals. Thus, the natural characteristic of enamel helps to eliminate the concern that the dominance of {100} faces may interrupt biopanning by competitively binding to phages. After four rounds of biopanning, the binding affinity of all 32 phage monoclones (named from E-1 to E-32, wherein E-1 from the original phage pool was used as control) to dental enamel surface was assessed by output/input efficiency, wherein the phage monoclone E-32 displaying amino acid sequence NNHYLRPR (EHBP) showed the highest affinity to enamel at an output/input ratio of 14.814 × 10^−7^, whereas E-1 displaying a random sequence WGNYAYK (RP) at a lower output/input ratio of 0.00035 × 10^−7^ and E-6 displaying amino acid sequence GQAGERK (ELBP) showed a moderate binding ability at a output/input ratio of 2.473 × 10^−7^ (Figure 1B). In contrast to the ELBP and RP, the phage displaying EHBP sequence exhibited the strongest affinity to enamel surface, indicating the corresponding peptide may have potential strong binding ability to amel surface.

**Figure 2:**
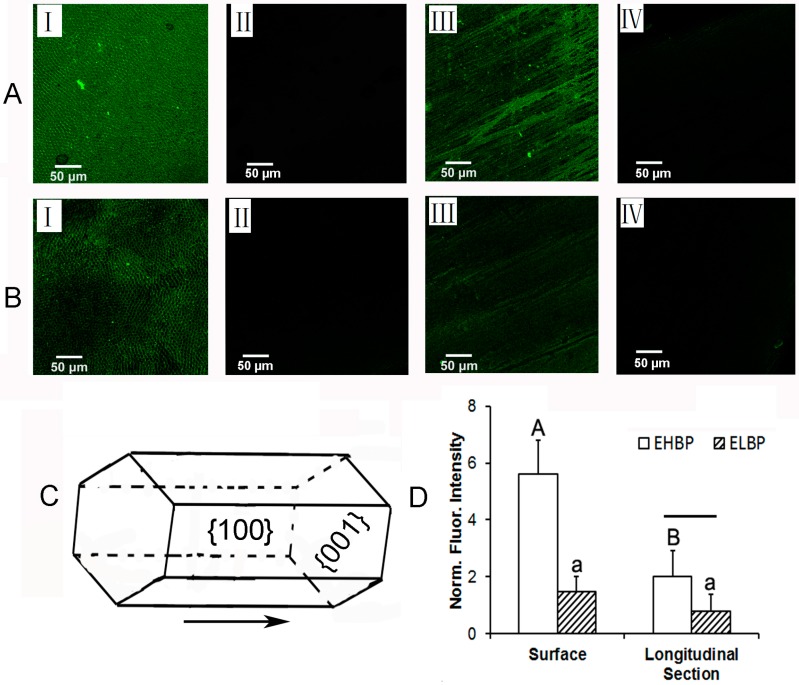
CLSM observation of FITC-labeled EHBP binding to enamel surface (**A-I**), and longitudinal section (**A-III**), as well as FITC-labeled ELBP binding to enamel surface (**B-I**) and longitudinal section (**B-III**). Fluoresce intensity of FITC without EHBP or ELBP as conjugates on the same enamel surface (**A-II**,**B-II**) or the longitudinal section (**A-IV**,**B-IV**) was served as control to normalize respective fluoresce intensity. Crystal {001} and {100} faces are illustrated in (**C**). For each parameter (upper case letters for EHBP and lower case letters for ELBP) in (**D**), groups with the same letters are not statistically significant (*p* > 0.05). For comparisons between EHBP and ELBP, groups connected with a bar are not statistically significant (*p* > 0.05).

To further verify the binding specificity of these peptides, the axis-specific binding ability has been studied by the CLSM analysis. CLSM images showed the fluoresce intensity of fluorescein isothiocyanate (FITC)-labeled EHBP binding to enamel surface (Figure 2A-I), and longitudinal section (Figure 2A-III), as well as FITC-labeled ELBP binding to enamel surface (Figure 2B-I) and longitudinal section (Figure 2B-III). Fluoresce intensity of FITC without EHBP or ELBP as conjugates on the same enamel surface (Figure 2A-II,B-II) or the longitudinal section (Figure 2A-IV,B-IV) was used as control to normalize respective fluoresce intensity. It was markedly found that EHBP fully covered both enamel rod area and inter-rod enamel area, demonstrating the specific affinity to enamel surface, whereas the ELBP only concentrated on the inter-rod area. The enamel rod and inter-rod enamel could be attributed to the crystal {001} and {100} faces, respectively, as illustrated in Figure 2C. To quantify the binding ability of peptide to enamel, the fluorescence intensity was visualized through FITC label and normalized with that of control group without phage added to the enamel surface (Figure 2A-II,B-II) or enamel longitudinal section (Figure 2A-IV,B-IV). It was found that the EHBP absorbing onto the enamel surface exhibited the significantly highest fluorescence intensity (5.6 ± 1.2), comparing to the intensity of EHBP to the longitudinal section (1.5 ± 0.9) (*p* < 0.05), as well as to the intensity of ELBP to enamel (1.5 ± 0.5) (*p* < 0.05). For the RP group, the fluorescence intensity could not be calculated for the weak signal of FITC-labeled RP to the enamel. The CLSM data validate the proposed hypothesis that peptide obtained from phage system against enamel {001} crystal faces would interact with {001} face in a face-specific style. This data was consistent with the output/input outcome, demonstrating the consensus sequence whatever in peptide or in phage clone play the same role in enamel face binding.

**Figure 3:**
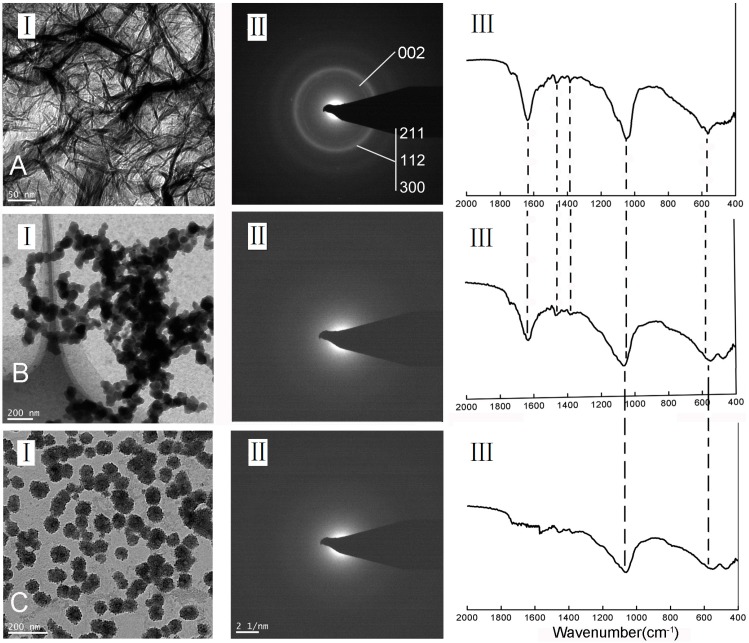
Unstained TEM images (**I**) and selected area electron diffraction (SAED) patterns (**II**), as well as the ATR-FTIR (**III**) of calcium phosphate minerals formed in the presence of 0.4 mM EHBP (**A**); 0.4 mM ELBP (**B**) and RP (**C**).

**Figure 4:**
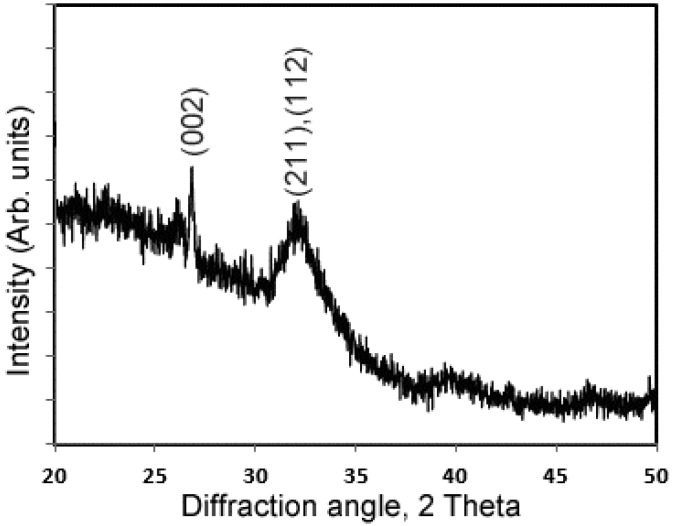
XRD profile of minerals formed in the presence of EHBP shows diffraction peaks at 2θ = 25.9° and 32.9° corresponding to the {002} and the {211}/{112} planes of hydroxyapatite.

Figure 3 shows the in vitro mineralization of EHBP (A), ELBP (B) and RP (C) characterized by TEM (I), SAED (II) and ATR-FTIR (III). After 4 h of mineralization, randomly oriented needle/plate-like aggregates were observed in the presence of EHBP, while SAED of crystals showed a ring pattern of 002 diffraction plane and a broad diffraction ring containing the 211, 112 and 300 triple diffraction planes, which confirmed the presence of apatite phase. IR spectra demonstrated typical υ_3_ PO_4_^3−^ peak (~1020 cm^−1^) and υ_4_ PO_4_^3−^ peaks (~601 cm^−1^ and ~563 cm^−1^) [47] along with amide I (~1635 cm^−1^) and amide II (1480 cm^−1^), which imply C=O and CN stretching of protein secondary structure [48]. For the mineral deposit in presence of ELBP, the IR spectra was similar to the EHBP group, which displays the typical PO_4_^3−^ group and amine group, however, the spherical particles were arranged in chain-like structures, with an amorphous SEAD ring. Regarding to RP group, isolated spherical particles with diameter of about 75 nm formed by ACP without incorporating any amide groups. The XRD profile of minerals formed in the presence of EHBP shows the marked fraction of crystalline phases of calcium phosphate (Figure 4), although the fraction was very low due to the limited sample obtained in current mineralization system setup.

The preferential binding of molecule to crystal face would inhibit the later crystal growth by binding to certain crystal face, which resulted in directed crystal orientation. For example of carbonated hydroxyapatite nanocrystals in enamel, the length to width aspect ratio reaches to as high as approximately 1000 [49], probably due to the morphology control of enamel extracellular matrix components (mainly amelogenins) [41]. Similarly in the vitro mineralization of EHBP, long needle/plate like HAp crystal was formed with high ratio of length to width, probably resulting from the inhibition effect of EHBP to HAp {001} face. With the decreasing affinity of ELBP and RP to HAp {001} face, only round and non-crystallized ACP particles precipitated. The ability of EHBP to accelerate the HAp nucleation through ACP transformation to crystalline HAp is consistent with recent findings from both natural protein and artificial peptides.

**Figure 5:**
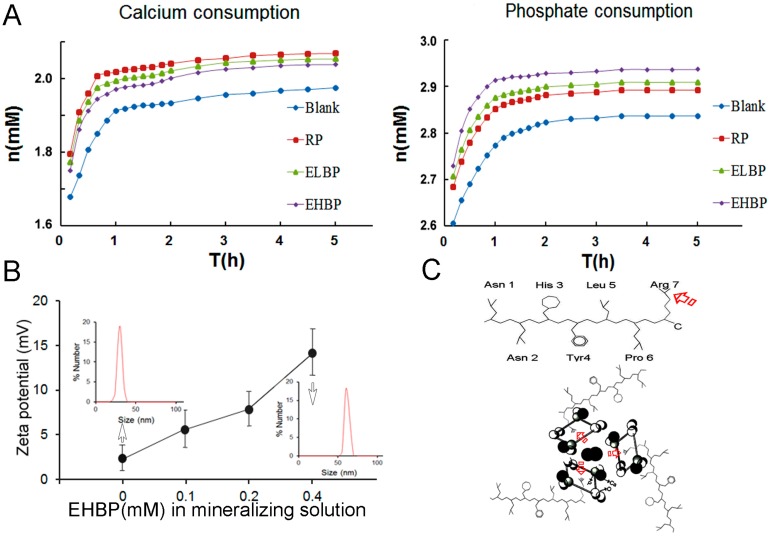
Rate of calcium ((**A**) left) and phosphate ((**A**) right) consumption in the presence of: no peptide, 0.4 mM RP, 0.4 mM ELBP and 0.4 mM EHBP. Zeta potentials of solution particles after EHBP added at different concentrations from 0 to 0.4 mM (**B**) and the change of particle distributions without EHBP ((**B**), inset) and in presence of 0.4 mM EHBP ((**B**), inset). The amino group arrangement of EHBP ((**C**) upper) and the interaction between EHBP and calcium phosphate clusters was schematically illustrated, where the arrow indicates the possible binding sites of EHBP to the PO4^3−^ group ((**C**), lower).

Figure 5A depicts the kinetics of mineral formation in the mineralizing solution in the presence of different peptides. Compared with solution with RP and ELBP, relatively low rate of calcium consumption and high rate of phosphate consumption in the mineralization solution with EHBP indicate the possible interaction of EHBP and phosphate ion. The effects of EHBP on the surface charge and particle size of ACP in the mineralization solution are displayed in Figure 5B. At the time point of measurement, the net surface charge of ACP was about 2.2 mV and the mean particle diameter was about 30 nm. After adding 0.4 mM EHBP, the surface charge and particle diameters were 14.1 mV and 58 nm, respectively.

It has been demonstrated that apatite formation does not occur directly by the association of ions from solution but rather proceeds through amorphous calcium phosphate (ACP) precursor phase, which have been found in bone [50], as well as in enamel [51]. Understanding interaction of biomolecules and amorphous calcium phosphate clusters existing in the mineralizing solution is critical to their applications in tissue engineering and biomimetic material synthesis. Compared to Glu (E) in ELBP, acidic residues, such as Asp (D) and Glu (E), are not found in EHBP, which is somewhat surprising because acidic amino acid-rich sequences were presumed to be binding sites of osteonectin (a natural HAp-binding protein) to HAp crystal [52,53,54]. In general, it is believed that negatively charged amino acids, containing carboxylate and phosphorylated residues, play a key role in controlling HAp mineralization. Nevertheless, increasing results reported by different researchers in recent years have shown that positively charged and polar amino acids can also interact with calcium phosphate clusters and can influence HAp particle size and crystallinity [28,55,56], implying the participation of both negatively and positively charged amino acids in regulation of biological process of HAp mineralization. Among the seven amino acids comprising EHBP, Pro (P) is an important structural amino acid in short peptide; His (H) and Arg (R) are basic residues. For a given peptide, the overall charge is mostly determined by the side chains of its constituent amino acid residues. The p*K*_a_ values of the two basic amino acids (H, R) in EHBP are 6.04 and 12.48, respectively. At pH 7.5, where the biopanning procedure was conducted, the R residues in EHBP is protonated and bears positive charges according to the Henderson-Hasselbalch equation, whereas H residue is not protonated. On the other hand, it is believed that the surface of HAp crystal is negatively charged possibly due to preferential surface migration of PO_4_^3−^ groups, which is considered to have a remarkable impact on organic adsorption [57]. Recently, the interaction of basic residues and PO_4_^3−^ ion of HAp mineral surface has been regarded as a contributing factor for the binding affinity of positively charged synthetic peptide to HAp surface, by an enhanced-sampling molecular simulation method [58]. In the current study, the complexion of PO_4_^3−^ ion and positively charged EHBP may dominate over that of Ca^2+^ ion and negatively charged amino acids in the binding process of synthetic peptide to HAp surface. The predominant adsorption of EHBP to the calcium phosphate clusters existing in the mineralizing solution may help to explain more rapid PO_4_^3−^ group consumption in contrast to the other two peptides, as well as control. Furthermore, the positive charged EHBP also increase the zeta potential of ACP precursor in the mineralizing solution. The higher concentration of EHBP, the more EHBP would be absorbed onto the calcium phosphate cluster to increase the surface charge and particle size in a dose-dependent manner (Figure 5B). From the dominance of basic amino acid residues in EHBP and the presence of negative charges on the surface of HAp at pH 7.5, we propose that electrostatic interactions between the C-terminal Arg and PO_4_^3−^ group play an important role in the EHBP-calcium phosphate clusters binding and afterwards HAp formation (Figure 5C). This is in line with recent works by Johromi, et al., who shows that positively charged amino acids (i.e., Arg) can interact with calcium phosphate prenuleation clusters and influence HAp particle size and crystallinity [55,56]. In addition, the C-terminal localization of Arg may allow for higher mobility of binding site and lower energy barrier of EHBP binding to calcium phosphate clusters or HAp surface [58].

## 4. Conclusions 

In this study, a new heptapeptide (i.e., EHBP) with high affinity to dental enamel surface was isolated through phage display. EHBP could bind to the dental enamel surface which was supposed to be arranged in HAp {001} faces. In the presence of EHBP, calcium phosphate aggregates were shown to crystalline into HAp, possibly through complexing PO_4_^3−^ ion by positively charged EHBP at neutral pH. Given the ability of EHBP binding to HAp {001} surface and directing calcium phosphate mineralization, this polypeptide may be used in the future as a mineralization directing motif in the hard tissue engineering [59]. We are currently working on testing the properties of a synthetic chimeric peptide containing self-assembly peptide (scaffold) and EHBP (nucleation site). Another attempt is to develop synthetic polypeptide antimicrobial activities with the enamel-binding capability for prevention of oral biofilm-related diseases.
